# Effect of low-intensity focused ultrasound on hippocampus of alcohol addicted mice: a preliminary study

**DOI:** 10.3389/fnins.2026.1789927

**Published:** 2026-04-17

**Authors:** Qian Wang, Xiaoqing Xiang, Lianjie Peng, Chunxia Liang, Qiong Li, Shuang Liang, Jianzhong Zou, Yanlin Chen

**Affiliations:** 1 Department of Pathology, Women and Children’s Hospital of Chongqing Medical University (Chongqing Health Center for Women and Children), Chongqing, China; 2 National Health Commission (NHC) Key Laboratory of Birth Defects and Reproductive Health, Chongqing, China; 3 Stomatology Hospital, School of Stomatology, Zhejiang University School of Medicine, Zhejiang Provincial Clinical Research Center for Oral Diseases, Key Laboratory of Oral Biomedical Research of Zhejiang Province, Hangzhou, Zhejiang, China; 4 State Key Laboratory of Ultrasound in Medicine and Engineering, College of Biomedical Engineering, Chongqing Medical University, Chongqing, China; 5 Chongqing Key Laboratory of Biomedical Engineering, Chongqing Medical University, Chongqing, China

**Keywords:** alcohol addiction, brain-derived neurotrophic factor, hippocampus, low-intensity focused ultrasound, neuromodulation

## Abstract

Alcohol addiction is a chronic relapsing brain disorder characterized by significant neurobiological changes, particularly within the hippocampus, which mediates emotional regulation and reward-seeking behavior. Previous studies have shown that alcohol-induced neuronal injury contributes to withdrawal-associated anxiety and persistent alcohol preference. This study investigated the therapeutic effects of low-intensity focused ultrasound (LIFU) on the hippocampus in a mouse model of alcohol addiction. Twenty-six male C57BL/6 mice were allocated to an alcohol-exposed group (*n* = 20) and a control group (*n* = 6). Following a 28-day modeling period, the alcohol group was randomly subdivided into a therapy group and a sham group. The therapy group received LIFU treatment, while the sham group underwent an identical procedure with the ultrasound transducer powered off. After seven days of treatment, the therapy group exhibited less severe anxiety symptoms upon alcohol withdrawal and a reduced preference for alcohol compared to the sham group. The brain-derived neurotrophic factor (BDNF) concentration was significantly lower in the therapy group than in the sham group, but did not differ significantly from the control group. Hippocampal HE staining revealed more pronounced degeneration and apoptosis of granule cells in the dentate gyrus (DG) region in the sham group relative to the therapy group. These preliminary findings suggest that LIFU may modulate alcohol addiction by mitigating hippocampal neuronal injury.

## 1 Introduction

Since the 1990s, a series of social and economic problems caused by excessive drinking have been increasing. Excessive drinking is associated with many diseases, such as cancer, cardiovascular disease, liver cirrhosis, fetal alcohol syndrome and neuropsychiatric diseases (Rehm et al., 2025). According to the survey, drinking became the seventh leading risk factor of death and disability of the global population in 2016 (Rehm et al., 2025; Griswold et al., 2018). Alcohol addiction, also known as alcohol dependence, is characterized by extremely high drinking motivation, difficulty in control, and a high susceptibility to relapse after limiting drinking (Beroun et al., 2018). Alcohol addiction is a serious consequence of long-term excessive drinking and simultaneously addiction aggravates drinking, which forms a vicious circle. It is repeatedly strengthened through the reward mechanism of the midbrain limbic dopamine system (Koob and Volkow, 2016; Wise and Robble, 2020; Lüscher and Malenka, 2011). The hippocampus is an important area of dopaminergic neurons in the limbic system, responsible for learning, memory and cognition (Deng et al., 2010). Studies have shown that the neurobiological mechanism of addiction is similar to the neurobiological mechanism of learning and memory (Hyman et al., 2006; Kelley, 2004; Volkow et al., 2014), that is, the hippocampus is involved in the formation of addiction (Wexler et al., 2001). Inhibition of synaptic transmission in granule cells in the dentate gyrus (DG) of the hippocampus can drive alcohol search and drinking, and alcohol can induce severe degeneration of hippocampal neurons (Beroun et al., 2018). Therefore, remodeling the morphology and function of hippocampal neurons in alcohol addicts may improve the degree of alcohol addiction. Brain-derived neurotrophic factor (BDNF), belonging to the neurotrophic protein family, is highly expressed in the hippocampus of rodents (Ernfors et al., 1990; Hohn et al., 1990). It promotes neuronal differentiation and proliferation, significantly influencing neuronal plasticity and the synthesis of other neurotrophic factors (Levi-Montalcini, 1987; Sendtner et al., 1992; Wang et al., 2022). In addition, it can also regulate the proliferation and survival of new neurons in the DG of the hippocampus (Foltran and Diaz, 2016). Therefore, adjusting the level of BDNF in the hippocampus of alcohol addicts can alleviate alcohol addiction by remodeling the morphology of the hippocampus.

Currently in Europe and the United States, the main treatment methods for alcohol addiction are psychosocial interventions, such as cognitive behavioral therapy, motivational interviews, and drug treatments, such as disulfiram, naltrexone, acamprosate. However, these treatments are poor in compliance, having many side effects. Drug treatments easily induce tolerance and have difficulty passing the blood–brain barrier (BBB) (Akbar et al., 2018; Wang et al., 2020). Some researchers directly injected exogenous BDNF into specific areas of the brain to achieve the therapeutic effect (Haun et al., 2018), but it will cause trauma, and there is a risk of infection, also the effective dose is difficult to control. Current research explores the use of electrical currents and magnetic fields to modulate the central nervous system for treating alcohol addiction, including methods such as deep brain stimulation (DBS), transcranial direct current stimulation (tDCS), and transcranial magnetic stimulation (TMS) (Henderson et al., 2010; Klauss et al., 2018). Although these methods are effective and easily penetrate the BBB, they still have many shortcomings, such as low spatial resolution and limited stimulation depth (Höppner et al., 2011). Therefore, new treatments for alcohol addiction still need to be explored. Low-intensity focused ultrasound (LIFU) combines high spatial specificity with deep penetration, propagating through tissue without causing damage. This profile gives it considerable potential for the clinical diagnosis and treatment of various diseases. Recent research has particularly highlighted its application to the central nervous system (Rezai et al., 2024; Martínez-Fernández et al., 2025; Hou et al., 2024; Yaakub et al., 2025; Xu et al., 2023; Zhu et al., 2023; Barksdale et al., 2025; Legon and Strohman, 2024). Compared with tDCS and TMS, LIFU has less tissue damage, stronger penetrating power, and better spatial resolution (Bystritsky et al., 2011; Rezai et al., 2024; Martínez-Fernández et al., 2025; Hou et al., 2024; Yaakub et al., 2025). It elevates BDNF levels in astrocytes via TrkB/PI3K/Akt and Ca/CaMKII signaling pathways, thereby influencing neuronal and other nervous system cell function and ameliorating post-withdrawal anxiety (Lin et al., 2015; Liu et al., 2017).

Based on the regulatory effect of LIFU on BDNF, this study investigates whether LIFU could reshape hippocampal neuronal morphology by modulating hippocampal BDNF, thereby reducing alcohol addiction severity and alleviating withdrawal symptoms following drinking cessation. We established a chronic alcohol addiction mouse model (Jiang et al., 2016), and applied LIFU to irradiate the hippocampus (Lin et al., 2015). The effect of LIFU on alcohol addiction was then evaluated by observing mouse behavior during withdrawal, measuring hippocampal BDNF concentration, and examining hippocampal tissue morphology.

## 2 Materials and methods

### 2.1 Laboratory animals

Thirty-eight healthy male C57BL/6 mice, 4–5 weeks old and free of disease, were provided by the Experimental Animal Center of Chongqing Medical University. 26 male mice were used in the addiction model (control group = 6, alcohol group = 20) and LIFU treatment (sham group = 9, therapy group = 9. During the treatment phase, one mouse in the sham group died from natural causes. To ensure a balanced experimental design, we randomly removed one mouse from the therapy group. The final sample size for both groups was *n* = 9), and 12 male mice were used to determine the appropriate duration of LIFU treatment (0 min group = 3, 5 min group = 3, 10 min group = 3, 15 min group = 3). All mice were housed in a specific pathogen-free (SPF) grade animal facility until 6 weeks of age, after which they were used in experiments. The animals were housed at a temperature controlled at (23 ± 2) °C and a humidity of (50 ± 10) %, with a 12-h light/12-h dark cycle, and had free access to water and feed.

### 2.2 Selection of LIFU irradiation intensity

Twelve normal mice were randomly divided into four groups (0 min group, 5 min group, 10 min group, and 15 min group), with three mice per group. LIFU was generated using a Model-CZG Ultrasound Therapeutic Device for Arthritis (Chongqing Haifu Medical Technology Co., Ltd., Chongqing, China) set to the following parameters: frequency 0.6 MHz ± 15%, power 0.6 W/cm^2^ ± 20%, and focal plane distance 28 mm ± 15%. Environmental conditions were kept consistent, and each group received daily irradiation for 0, 5, 10, or 15 min at a fixed time over 7 consecutive days. Prior to each session, mice were anesthetized with isoflurane, the head hair was shaved, and ultrasound coupling gel was applied to the exposed skin. The ultrasound probe (diameter 2 cm) was positioned over the hippocampal projection area on the skull, centered midway between the ears, corresponding to a circular treatment area of *π* cm^2^. To ensure the comfort of the mice during this process, they were placed in an airtight container and anesthetically induced with isoflurane at 3% for 3 min. Afterward, they were fitted into a nose mask and the isoflurane was lowered to 1.5% for the remainder of the process, which lasted for an additional 20 min.

After the 7-day irradiation, the 4 groups of mice were sacrificed by neck dislocation. We measured the level of BDNF in their hippocampus. And then we chose the most appropriate irradiation intensity applied to irradiation on alcohol addiction model, based on the level of BDNF protein in the hippocampus of the mice. Procedure: After using cervical dislocation to euthanize the mice, brain tissues were dissected from the skull and placed on an ice-cold surface. Using sterile forceps, the left and right hippocampal tissues were isolated, transferred to a 1.5 mL sterile tube, and stored at −80 °C. For analysis, the hippocampal samples were thawed and processed into a 10% tissue homogenate. Each sample was rinsed with normal saline, blotted dry on absorbent paper, and weighed before being homogenized for 15 min in a chilled glass homogenizer with the appropriate volume of saline. The resulting homogenate was centrifuged at 4 °C (3,000 rpm) for 15 min to collect the supernatant. All subsequent steps followed the manufacturer’s protocol for the commercial mouse BDNF ELISA kit (Shanghai Xi tang Biotechnology Co., Ltd., Shanghai, China), with absorbance measured using an ELX800 microplate reader (Gene Company Limited, Shanghai, China).

### 2.3 Establishment of mouse models of alcohol addiction

Twenty-six mice were randomly assigned to either a control group (free water intake, *n* = 6) or an alcohol group (*n* = 20). The alcohol group underwent a 28-day alcohol addiction modeling procedure. Specific details regarding alcohol concentration and administration method were as follows: Following previous two-bottle free-choice experiments (Belknap et al., 1993; Rhodes et al., 2005; Crabbe et al., 2010; Juarez et al., 2017), 6-week-old male C57BL/6 mice were allowed voluntary access to water and gradually increasing concentrations of alcohol (alcohol groups received solutions with volume ratios of 5, 10, 20, and 35% during weeks 1, 2, 3, and 4, respectively). The control group received standard double-distilled water throughout the experiment, with all conditions kept constant except for the feeding and modeling protocol. The liquor used was obtained from Chongqing Jiangjin Winery Co., Ltd. (Chongqing, China). The purchased liquor had an alcohol content of 50%, which was diluted with double-distilled water to concentrations of 5, 10, 20, and 35% for use.

After the 28-day modeling period, we gave 12 h of abstinence from water or alcohol to both the control group and the alcohol group. Then the two groups were tested to detect whether mouse models of alcohol addiction had been successfully established. Specifically, we tested the two groups for alcohol preference test. At the beginning of the experiment, keeping the experiment environment quiet, we gave each mouse a bottle of 10% alcohol solution and a bottle of double-distilled water (5 mL each) respectively and exchanged the positions of the alcohol bottle and the water bottle after 30 min to eliminate the impact of position preference. And then we, respectively, recorded the consumption of alcohol solution and double distilled water in 1 h and calculated the alcohol consumption rate which indicates the degree of alcohol preference [alcohol consumption rate = alcohol solution consumption/(alcohol solution consumption + double distilled water consumption) × 100%]. To ensure the reliability of the addiction model and the homogeneity of the experimental groups, we applied a quantitative screening criterion based on the Alcohol Preference Test on day 28. Specifically, only mice exhibiting an alcohol preference ratio of greater than 35% were considered to have successfully established alcohol dependence and were included in the subsequent LIFU intervention (Becker, 2008; Xu et al., 2023; Belknap et al., 1993).

After the alcohol preference test, all mice underwent an open field test and forced swimming test to assess the overall negative affective state (including both depressive-like and anxiety-like elements) induced by alcohol withdrawal. The open field test was conducted in a dark, quiet room using a square arena (45 × 45 × 45 cm) subdivided into 16 equal squares, with the four central squares defined as the center zone. At the start of the trial, each mouse was gently placed by the tail into a corner of the arena and allowed to explore freely for 1 min. Subsequently, its movement was recorded for 5 min using Smart 3.0 behavioral analysis software (Shenzhen Reward Life Technology Co., Ltd., Shenzhen, China) to obtain the total distance traveled, the time and distance spent in the center zone as well as the immobility time. Following each test, mice were removed, and the arena was thoroughly cleaned inside and out with 75% ethanol to eliminate olfactory cues for the next subject. The center time ratio was calculated as (time in center/total time) × 100%, and the center distance ratio as (distance in center/total distance) × 100%. Then the two groups of mice were subjected to a forced swimming test. The interval between the two behavioral tests was 4 h. The operation method of the forced swimming test was as follows. Firstly, we filled a water tank with a height of 30 cm and a diameter of 20 cm to a height of 15 cm, and the water temperature was maintained at 22–25 °C. Then we gently grasped the tail of the mouse and placed it into the water tank, observed for 6 min, and recorded the immobility time after 4 min. The standard of static state is that there is no active, spontaneous escape behavior, and only the movement of the tiny limbs with the head floating on the water.

### 2.4 LIFU irradiation on alcohol addiction model

Based on the previous experimental results, an irradiation time of 15 min was selected for irradiating the model. The control group continued to have free access to water. Mice in the alcohol group were randomly assigned to either the therapy group or the sham group (10 mice per group, but during the treatment phase, one mouse in the sham group died from natural causes. To ensure a balanced experimental design, we randomly removed one mouse from the therapy group. The final sample size for both groups was *n* = 9). The therapy group then received LIFU irradiation targeted at the hippocampus for the appropriate duration (power: 0.6 W/cm^2^ ± 20%, focal plane distance: 28 mm ± 15%, 15 min/d for 7 consecutive days), following the specific method described previously. Throughout this 7-day irradiation period, both the treatment and sham groups were maintained on a 5% alcohol solution. All other conditions were kept consistent except for the radiation intervention.

After the 7-day irradiation period, both groups were deprived of alcohol for 12 h. After 12 h, the alcohol preference test and a series of behavioral tests, including the OFT, FST and elevated plus maze (EPM) test, were performed in both the therapy and sham groups, with a 4-h interval between consecutive tests. The alcohol preference test reflects the difference between the degree of psychological craving for alcohol and the degree of drug-seeking in each group of mice after the LIFU intervention. The operation of the alcohol preference test, the open field test and the forced swimming test were the same as those described above.

The operation method of the EPM was as follows. The mouse was removed from its cage and placed at the junction of the open and closed arms of the EPM (facing the open arm); the apparatus (homemade) consisted of two open arms (30 cm × 6 cm × 15 cm) and two closed arms (30 cm × 6 cm × 15 cm) intersecting at a central platform (5 cm × 5 cm) elevated 50 cm above the ground. The animal was allowed to explore freely for 5 min. The time spent in the open arms and closed arms were recorded separately.

After the behavioral experiments were completed, three mice each were randomly selected from the control, therapy, and sham groups to make whole brain slices for histological observation. First, the mice were anesthetized with isoflurane following the specific method described previously and systemically fixed via cardiac perfusion. The chest was opened to expose the heart, and the left ventricle was perfused with saline; an incision was made through the liver to drain most of the blood before switching to 4% paraformaldehyde until the tissues hardened. The whole brain was then rapidly extracted and post-fixed in 4% paraformaldehyde for paraffin embedding. Paraffin sections were prepared by the Laboratory of Ultrasound Research Institute of Chongqing Medical University and stained with hematoxylin and eosin (HE). Hippocampal cell morphology in the CA1, CA3, and DG regions was examined under a light microscope (BX53; Olympus, Tokyo, Japan). The remaining mice in each group were euthanized by cervical dislocation, after which the hippocampus from each hemisphere was dissected for subsequent BDNF quantification, the operation procedure as described above.

### 2.5 Statistical methods

SPSS version 26.0 (IBM Corp., Armonk, NY, United States) was used for statistical processing. Measurement data were expressed as mean ± SD. The alcohol preference test and behavioral tests were performed using *t-tests* to examine the data. ELISA data for BDNF levels in the hippocampus were analyzed using one-way ANOVA followed by Dunnett’s multiple comparisons test. *p* < 0.05 was considered statistically significant.

## 3 Results

### 3.1 Optimization of LIFU parameters: duration-dependent effects on hippocampal BDNF

**Figure 1:**
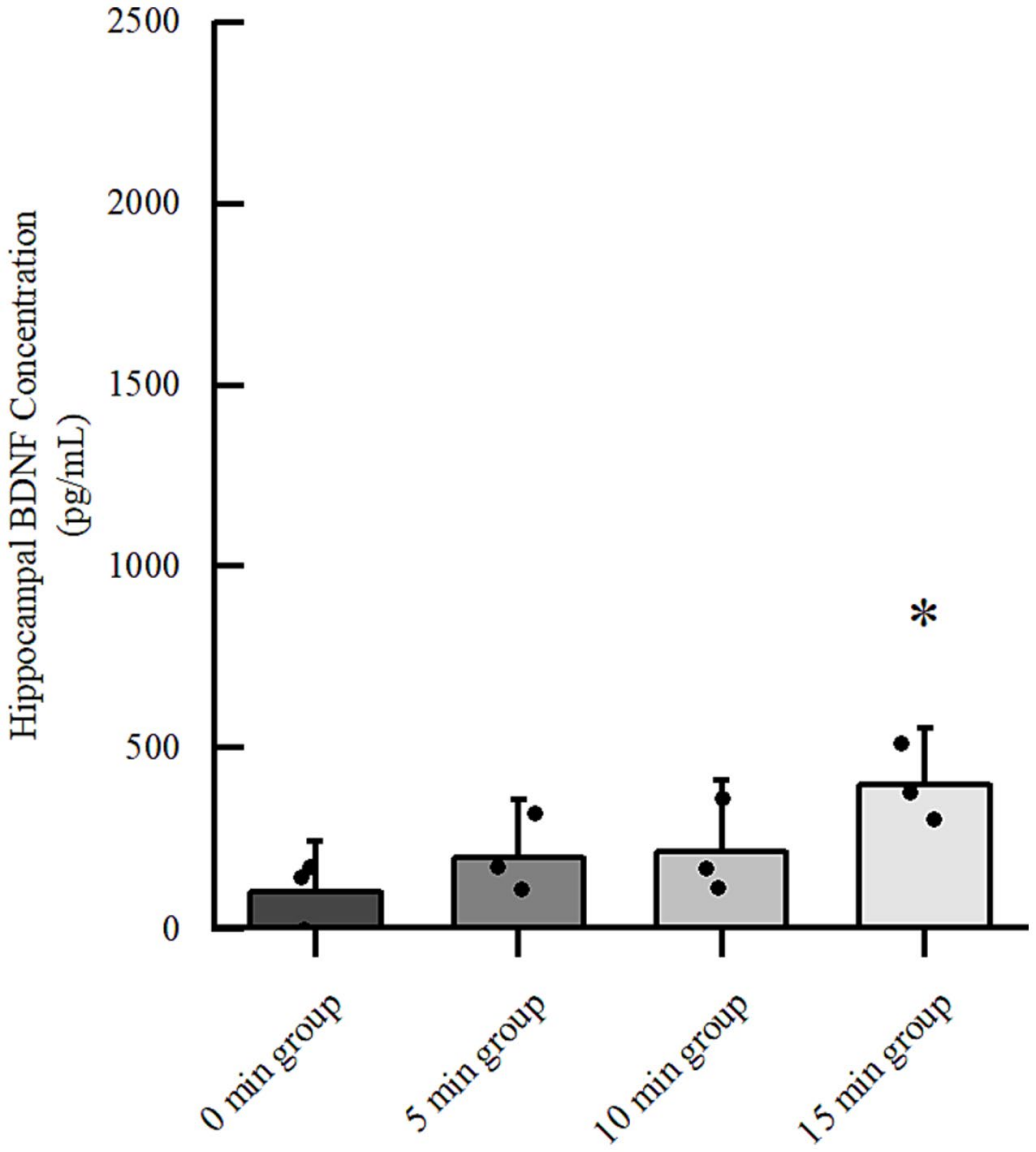
Effects of different durations of LIFU irradiation on BDNF concentration in the hippocampus of normal mice. *p < 0.05 vs 0 min group.

To determine the optimal irradiation duration for neuromodulation, we assessed hippocampal BDNF concentrations in control mice after 7 days of LIFU treatment at various durations (0, 5, 10, and 15 min). BDNF levels exhibited a duration-dependent increasing trend: 103.60 ± 92.99 pg./mL for 0 min, 199.00 ± 106.49 pg./mL for 5 min, 213.74 ± 130.59 pg./mL for 10 min, and 397.34 ± 104.02 pg./mL for 15 min. Notably, while the 5 and 10 min groups showed upward trends without reaching statistical significance, the 15 min group displayed a significant elevation in BDNF compared to the 0 min group (Figure 1, *p* < 0.05). Consequently, the 15-min duration was selected as the optimal protocol for subsequent therapeutic experiments.

### 3.2 Verification of the alcohol addiction mouse model

#### 3.2.1 Alcohol preference and seeking behavior

**Figure 2:**
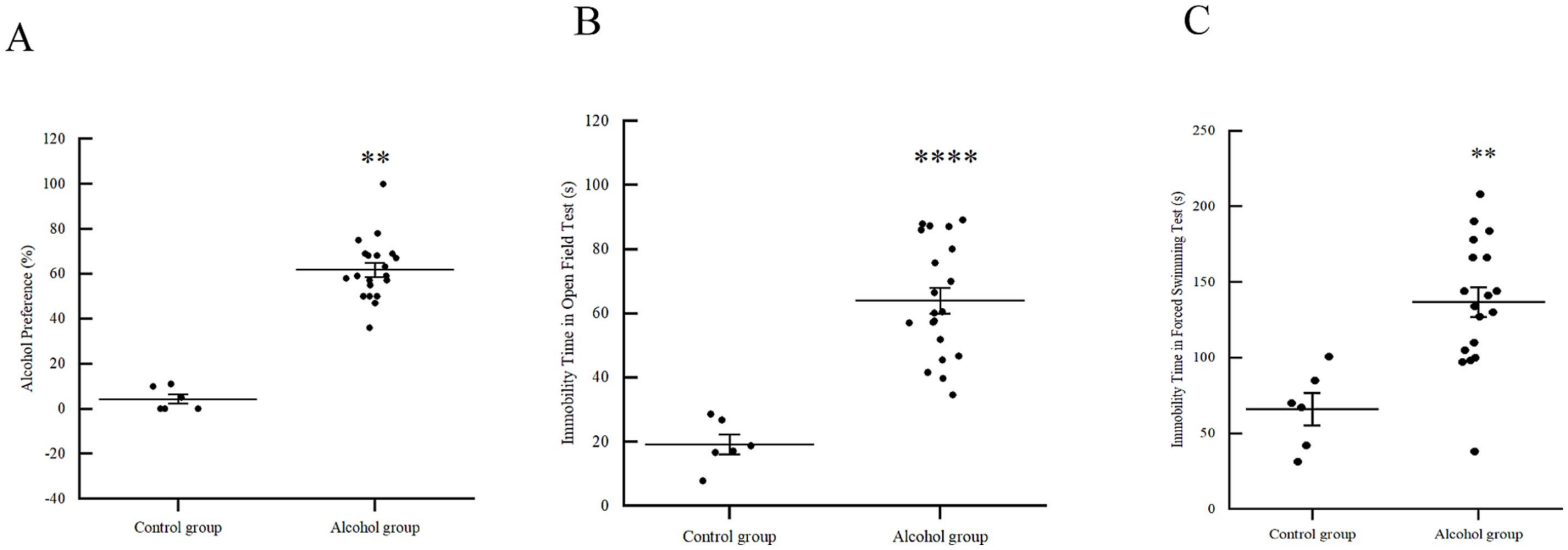
Effects of alcohol addiction on mice. **(A)** The effects of alcohol consumption on alcohol preference test. *p* < 0.01 vs. control group. **(B)** The effects of alcohol consumption on open field test. *p* < 0.0001 vs. control group. **(C)** The effects of alcohol consumption on forced swimming test. *p* < 0.01 vs. control group.

After a 28-day modeling period, the alcohol group exhibited a marked increase in alcohol preference. The preference rate reached (61.76 ± 13.63)%, which was significantly higher than that of the control group (4.33 ± 5.20)% (Figure 2A, *p* < 0.01). Importantly, all mice in the alcohol group maintained a preference rate exceeding 35%, confirming the successful establishment of the alcohol addiction model.

#### 3.2.2 Assessment of negative affective states (OFT and FST)

Behavioral assessments revealed significant negative affective states in addicted mice. In the OFT, although central time and distance ratios showed no significant differences (Table 1), the immobility time was dramatically increased in the alcohol group (64.05 ± 17.94 s) compared to the control group (19.26 ± 7.58 s) (Figure 2B, *p* < 0.0001). Similarly, in the FST, the absolute immobility time of the alcohol group (136.65 ± 41.80 s) was significantly higher than that of the control group (65.97 ± 26.00 s) (Figure 2C, *p* < 0.01). Together, these results indicate that chronic alcohol consumption induces significant anxiety-like and depressive-like behaviors during the early withdrawal phase.

**Table 1:** The effects of alcohol consumption on the open-field test.

Items	Control	Alco	*p*-value
Number of animals	6	20	
Time in center zone (%)	5.13 ± 3.76	4.65 ± 3.40	>0.05
Distance in center zone (%)	9.73 ± 4.13	11.85 ± 4.89	>0.05

### 3.3 Therapeutic effects of LIFU on alcohol-addicted mice

#### 3.3.1 Mitigation of alcohol seeking and negative affect

**Figure 3:**
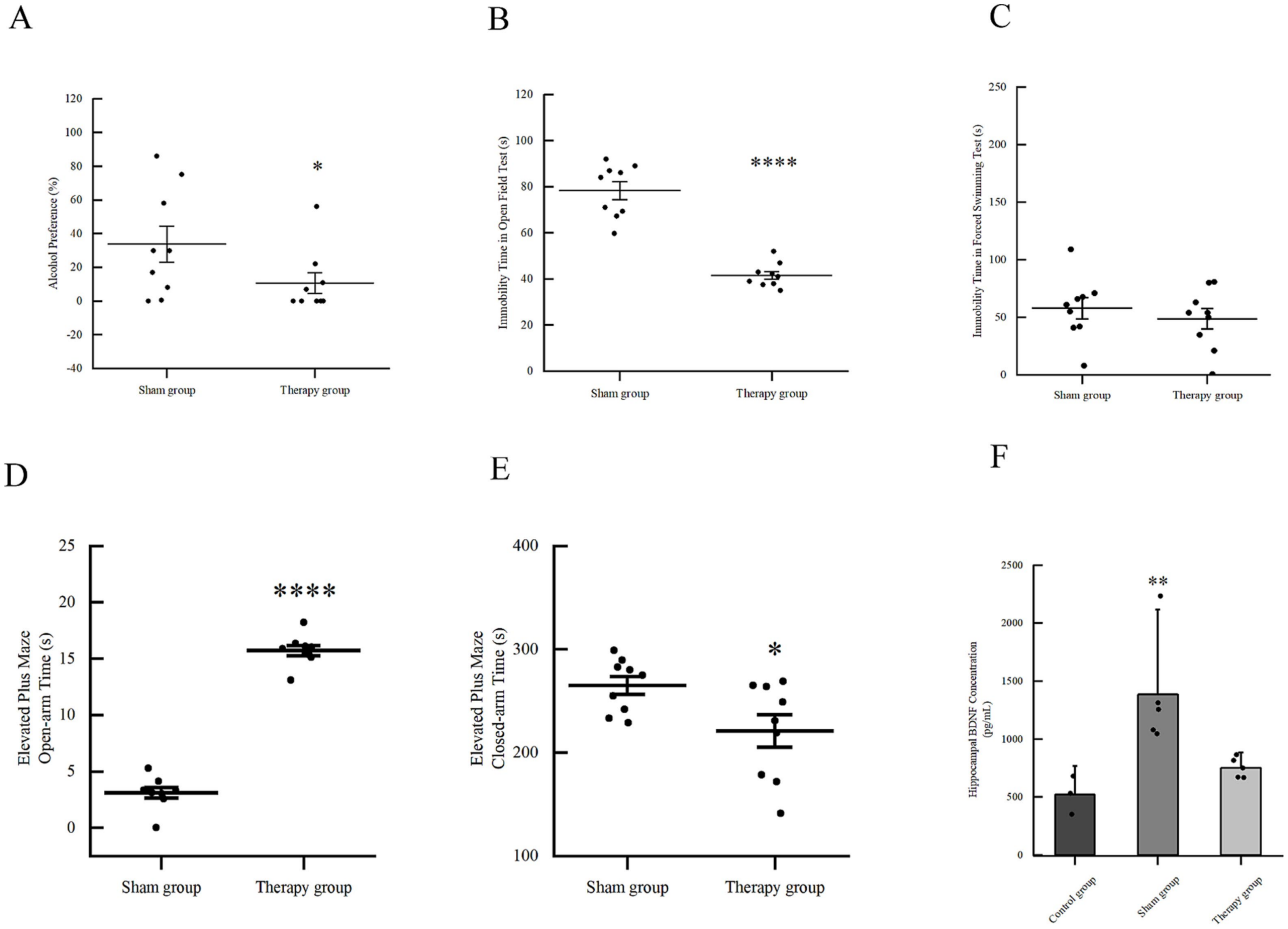
Effects of LIFU on mice with alcohol addiction. **(A)** The effects of LIFU for alcohol addiction model on alcohol preference test. ^*^p < 0.05 vs. sham group. **(B)** The effects of LIFU for alcohol addiction model on open field test. ****p < 0.0001 vs. sham group. **(C)** The effects of LIFU for alcohol addiction model on forced swimming test. There is no statistical difference between the sham group and the therapy group. **(D)** The effects of LIFU for alcohol addiction model on elevated plus maze test (time in open arms). ****p < 0.0001 vs. sham group. **(E)** The effects of LIFU for alcohol addiction model on elevated plus maze test (time in closed arms). *p < 0.05 vs. sham group. **(F)** ELISA analysis for BDNF concentration in hippocampus of LIFU for alcohol addiction model. **p < 0.01 vs. both control and therapy groups.

Following 7 days of LIFU intervention, the alcohol preference rate in the therapy group (10.67 ± 18.59) % was significantly lower than that of the sham group (40.44 ± 30.44)% (Figures 3A, *p* < 0.05). Behavioral recovery was further evidenced in the OFT and EPM. In the OFT, LIFU significantly reduced the immobility time to (41.64 ± 5.27) s, compared to (78.38 ± 11.60) s in the sham group (Figure 3B, *p* < 0.0001). However, there was no statistically significant difference between the two groups for the central time ratio and central distance ratio (Table 2). In the EPM, LIFU treatment significantly increased the time spent in open arms (15.72 ± 1.34 s vs. 3.12 ± 1.50 s, *p* < 0.001) and decreased the time in closed arms (220.95 ± 46.88 s vs. 265.09 ± 25.83 s, *p* < 0.05) (Figures 3D,E). LIFU treatment slightly reduced the FST immobility time (44.42 ± 29.13 s) relative to the sham group (65.75 ± 38.37 s), though without statistical significance (Figure 3C, *p* < 0.05). These findings collectively demonstrate that LIFU effectively modulated alcohol-seeking behavior and associated anxiety-like symptoms.

**Table 2:** The effects of LIFU for alcohol addiction model on the open-field test.

Items	Sham	Therapy	*p*-value
Number of animals	9	9	
Time in center zone (%)	2.76 ± 1.56	3.37 ± 1.79	>0.05
Distance in center zone (%)	12.17 ± 5.71	15.70 ± 2.67	>0.05

#### 3.3.2 Homeostatic regulation of hippocampal BDNF

ELISA analysis revealed distinct BDNF dynamics. In the sham group (alcohol withdrawal without LIFU), hippocampal BDNF levels surged to (1385.35 ± 487.84) pg./mL, which is consistent with the relevant literature we reviewed (McGough et al., 2004; Crews et al., 2000; Heberlein et al., 2016; Portelli et al., 2020). This significantly exceeded the level of control group (520.65 ± 164.23) pg./mL (Figure 3F, *p* < 0.01). This suggests that acute withdrawal triggers a pathological compensatory spike in BDNF. However, LIFU treatment significantly modulated this response, reducing BDNF levels to (752.48 ± 87.08) pg./mL, effectively restoring them toward a normal physiological range.

#### 3.3.3 Histologic analysis

**Figure 4:**
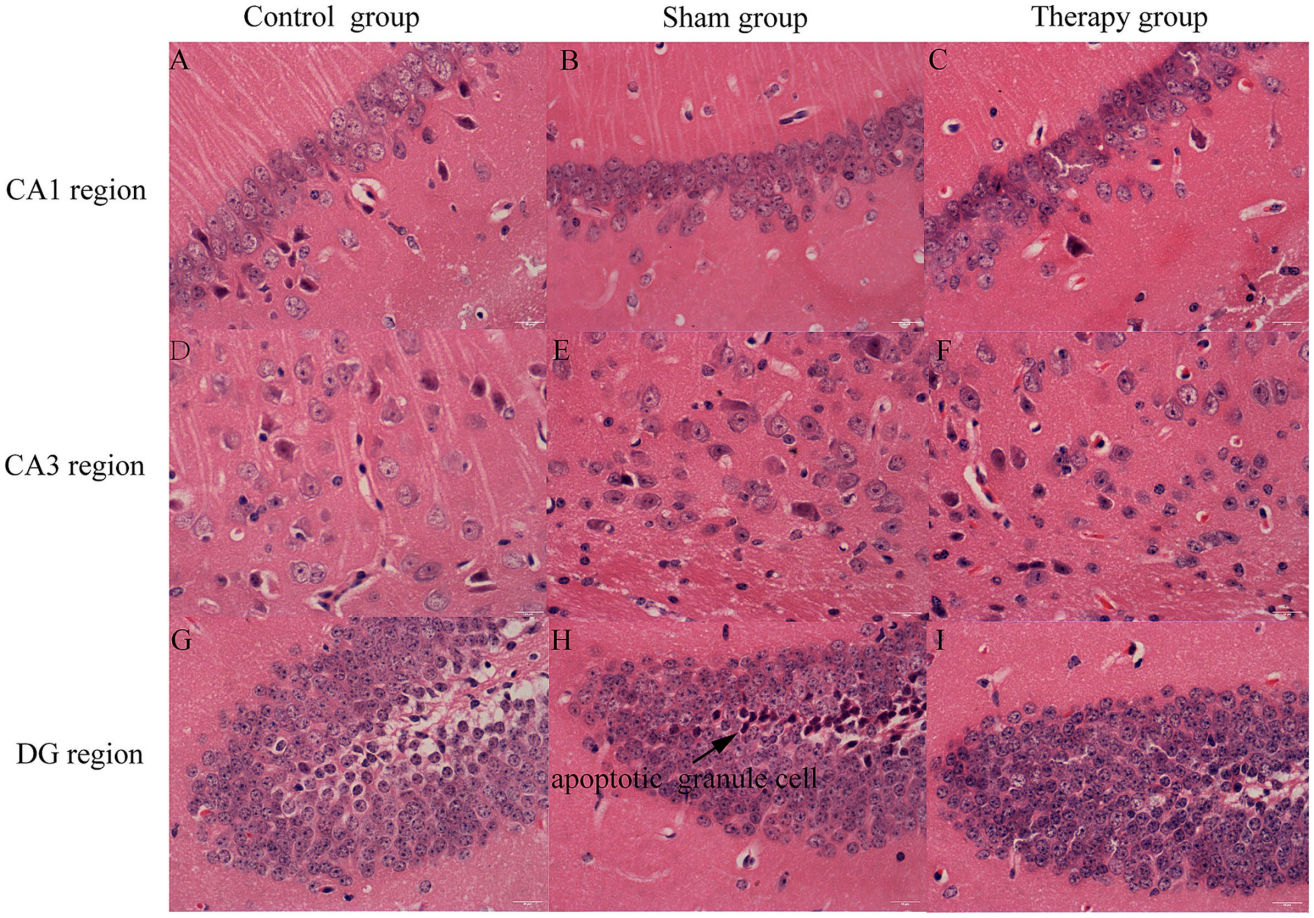
Histologic analysis of the CA1, CA3, and DG regions in hippocampus. **(A)** CA1 region of C57 mouse in the control group (hematoxylin and eosin, ×400). **(B)** CA1 region of C57 mouse in the sham group (hematoxylin and eosin, ×400). **(C)** CA1 region of C57 mouse in the therapy group (hematoxylin and eosin, ×400). **(D)** CA3 region of C57 mouse in the control group (hematoxylin and eosin, ×400). **(E)** CA3 region of C57 mouse in the sham group (hematoxylin and eosin, ×400). **(F)** CA3 region of C57 mouse in the therapy group (hematoxylin and eosin, ×400). **(G)** DG region of C57 mouse in the control group (hematoxylin and eosin, ×400). **(H)** DG region of C57 mouse in the sham group (hematoxylin and eosin, ×400). **(I)** DG region of C57 mouse in the therapy group (hematoxylin and eosin, ×400).

Histological examination highlighted subregion-specific neuroprotection by LIFU. Pyramidal cells in the CA1 and CA3 regions and granule cells in the DG region of the control group appeared large, structurally intact, and arranged in an orderly, compact manner, with only occasional apoptotic cells observed. In the sham group, pyramidal cells in CA1 exhibited no obvious degeneration. Pyramidal cells in the CA3 region and granule cells in the DG region, however, displayed increased apoptosis, characterized by deeply stained cytoplasm and reduced cell volume, though their overall arrangement remained largely normal. Within the therapy group, the number of apoptotic pyramidal cells in CA3 remained relatively high, but apoptotic granule cells in DG were significantly fewer than in the sham group (Figure 4). This suggests that LIFU-mediated BDNF regulation specifically preserves the structural integrity of the hippocampal dentate gyrus.

## 4 Discussion

Alcohol is a pervasive addictive substance globally. Clinical research indicates that alcohol dependence leads to severe withdrawal symptoms, including intense drug-seeking behavior, anxiety, and depression (Becker, 2008). These affective states are pivotal in the persistence of addiction; therefore, alleviating anxiety and depression-like behaviors serves as a key indicator of therapeutic efficacy (Beroun et al., 2018; Kang et al., 2018). In the present study, LIFU intervention significantly modulated alcohol-seeking behavior and attenuated the negative affective states induced by withdrawal, demonstrating its potential as a non-invasive neuromodulatory tool for addiction.

A striking finding in our research was the differential expression of BDNF across groups. Previous studies have shown that BDNF expression undergoes a dynamic shift—initially upregulating during prolonged drinking before gradually declining (MacLennan et al., 1995; McGough et al., 2004). In our study, BDNF levels in the untreated group were significantly higher than those in both the control and ultrasound-treated groups. We speculate that the untreated group, being in the acute withdrawal phase, experienced severe hippocampal injury characterized by excitotoxicity, oxidative stress, and neuroinflammation (Crews et al., 2000). This pathological state may trigger a compensatory surge in BDNF as a stress response to combat acute injury (Huang et al., 2008). Notably, our results show that LIFU restored BDNF to near-normal levels. While LIFU is traditionally known to upregulate BDNF in neurodegenerative models like Alzheimer’s or stroke (Lin et al., 2015; Rezayat and Toostani, 2016; Chen et al., 2019), its biological response is highly context-dependent.

Our findings align with Jia-mou et al. (2009), who observed BDNF downregulation following specific ultrasound protocols, and Phan et al. (2025), who demonstrated that theta-burst ultrasound significantly reduced astrocytic BDNF in a pain model. Together, these data suggest that LIFU exerts a homeostatic regulatory effect by normalizing elevated BDNF levels in hyperexcitable states, rather than inducing a simple linear increase.

Histopathological analysis further supported the protective role of LIFU. Untreated alcohol-addicted mice exhibited significant degeneration and apoptosis of DG granule cells. This damage likely stems from acute withdrawal-induced BDNF overexpression, which triggers the TrkB signaling cascade, leading to intracellular Ca^2+^ overload and subsequent neurotoxicity (Logrip et al., 2008). In contrast, the DG morphology in the ultrasound-treated group remained comparable to the control group. This suggests that by regulating BDNF to physiological levels, LIFU effectively prevented the downstream calcium-mediated apoptotic cascade, thereby preserving hippocampal structural integrity (Foltran and Diaz, 2016).

This study has several limitations. First, while we characterized BDNF levels and morphology, the specific downstream signaling pathways remain to be elucidated. Second, the use of whole-hippocampus irradiation precluded the observation of hemispheric disparities; future research will employ hemisphere-specific stimulation to enable intra-subject comparisons. Additionally, logistical constraints during the COVID-19 pandemic prevented the collection of blood alcohol concentration (BAC) data. Finally, given the modest sample size, larger cohorts are warranted to optimize the clinical translation of LIFU for addiction recovery.

In summary, hippocampal LIFU irradiation effectively regulates alcohol withdrawal symptoms and alcohol-seeking behavior in mice, likely through the homeostatic modulation of BDNF levels. Given that drug addiction is reinforced through the reward circuitry of the limbic dopamine system (Wexler et al., 2001; Koob and Volkow, 2016; Wise and Robble, 2020; Lüscher and Malenka, 2011), the neuromodulatory capacity of LIFU provides a promising strategy for treating various substance use disorders.

## 5 Conclusion

Studies in animal models have confirmed that LIFU therapy produces therapeutic effects on alcohol addiction in C57BL/6 mice, as evidenced by a reduction in apoptotic granule cells within the DG region post-treatment. Consequently, LIFU therapy has been shown to regulate alcohol addiction withdrawal symptoms and addiction severity by improving the hippocampal neuronal morphology through the modulation of local BDNF levels. It should be noted that the present work is a preliminary study with a relatively small sample size. Future studies with larger cohorts are warranted to validate these findings and further explore the underlying mechanisms.

## Data Availability

The original contributions presented in the study are included in the article/supplementary material, further inquiries can be directed to the corresponding authors.
